# Construction of lignan glycosides biosynthetic network in *Escherichia coli* using mutltienzyme modules

**DOI:** 10.1186/s12934-024-02467-1

**Published:** 2024-07-05

**Authors:** Yuqi Qiao, Doudou Huang, Yajing Li, Songfan Jiang, Xiao Chen, Junfeng Chen, Ying Xiao, Wansheng Chen

**Affiliations:** 1 Research and Development Center of Chinese Medicine Resources and Biotechnology, The Ministry of Education (MOE) Key Laboratory for Standardization of Chinese Medicines, Institute of Chinese Materia Medica, Shanghai University of Traditional Chinese Medicine, Shanghai, 201203 China; 2 Department of Pharmacy, Changzheng Hospital, Second Military Medical University, Shanghai, 200003 China; 3 School of Traditional Chinese Medicine, Shanghai University of Traditional Chinese Medicine, Shanghai, 201203 China; 4 Shanghai Key Laboratory of Plant Molecular Sciences, College of Life Sciences, Shanghai Normal University, Shanghai, 200234 China

**Keywords:** Lignans, Heterologous biosynthesis, *Escherichia coli*, Module assembly

## Abstract

**Background:**

Due to the complexity of the metabolic pathway network of active ingredients, precise targeted synthesis of any active ingredient on a synthetic network is a huge challenge. Based on a complete analysis of the active ingredient pathway in a species, this goal can be achieved by elucidating the functional differences of each enzyme in the pathway and achieving this goal through different combinations. Lignans are a class of phytoestrogens that are present abundantly in plants and play a role in various physiological activities of plants due to their structural diversity. In addition, lignans offer various medicinal benefits to humans. Despite their value, the low concentration of lignans in plants limits their extraction and utilization. Recently, synthetic biology approaches have been explored for lignan production, but achieving the synthesis of most lignans, especially the more valuable lignan glycosides, across the entire synthetic network remains incomplete.

**Results:**

By evaluating various gene construction methods and sequences, we determined that the pCDF-Duet-Prx02-*Ps*VAO gene construction was the most effective for the production of (+)-pinoresinol, yielding up to 698.9 mg/L after shake-flask fermentation. Based on the stable production of (+)-pinoresinol, we synthesized downstream metabolites in vivo. By comparing different fermentation methods, including “one-cell, one-pot” and “multicellular one-pot”, we determined that the “multicellular one-pot” method was more effective for producing (+)-lariciresinol, (-)-secoisolariciresinol, (-)-matairesinol, and their glycoside products. The “multicellular one-pot” fermentation yielded 434.08 mg/L of (+)-lariciresinol, 96.81 mg/L of (-)-secoisolariciresinol, and 45.14 mg/L of (-)-matairesinol. Subsequently, ultilizing the strict substrate recognition pecificities of UDP-glycosyltransferase (UGT) incorporating the native uridine diphosphate glucose (UDPG) Module for in vivo synthesis of glycoside products resulted in the following yields: (+)-pinoresinol glucoside: 1.71 mg/L, (+)-lariciresinol-4-*O*-d-glucopyranoside: 1.3 mg/L, (+)-lariciresinol-4’-*O*-d-glucopyranoside: 836 µg/L, (-)-secoisolariciresinol monoglucoside: 103.77 µg/L, (-)-matairesinol-4-*O*-d-glucopyranoside: 86.79 µg/L, and (-)-matairesinol-4’-*O*-d-glucopyranoside: 74.5 µg/L.

**Conclusions:**

By using various construction and fermentation methods, we successfully synthesized 10 products of the lignan pathway in *Isatis indigotica* Fort in *Escherichia coli*, with eugenol as substrate. Additionally, we obtained a diverse range of lignan products by combining different modules, setting a foundation for future high-yield lignan production.

**Supplementary Information:**

The online version contains supplementary material available at 10.1186/s12934-024-02467-1.

## Background

With the complete elucidation of the biosynthetic pathways, many active ingredients in medicinal plants have been efficiently produced heterologously through metabolic engineering approaches. For example, ginsenosides Rh2 or Rg3 have been produced from glucose by constructing yeast cell factories [1]. By constructing recombinant *Escherichia coli*, the precursor taxadien-5α-ol of the anticancer drug paclitaxel has been produced heterologously [2]. However, current reports have focused on the synthesis of a single component, while many synthetic pathways contain multiple active ingredients. The challenge lies in how to accurately and efficiently achieve the targeted enrichment of a series of active ingredients on the same metabolic network, which is highly challenging. Lignans constitute a broad category of phytoestrogens found in plants and play a crucial role as polyphenolic compounds [3]. Structurally, lignans consist of two phenylpropane units connected by a C_6_-C_3_ bond at the β-β′ position of the propyl side chain. This configuration facilitates stereoselective oxidative coupling [4] and allows for various aromatic ring substitution patterns [5]. Lignans are found across a spectrum of species in the plant kingdom, including nonvascular bryophytes, such as *Anthoceros punctatus* (hornwort) [6], *Lepicolea ochroleuca* (liverwort) [7], and *Bazzania trilobata* (three-lobed Bazzania) [8], as well as pteridophytes such as ferns [9], gymnosperms, and angiosperms.

Lignans exhibit structural diversity, which is linked to their various biological activities in plants, including antioxidant, antibacterial, antifungal, and insecticidal effects. Moreover, lignans exert diverse effects upon entering human body. They are present as vital nutrients in oilseeds (e.g., flaxseed, sesame, and sunflower seeds), whole grains (e.g., wheat, oats, rye, and barley), legumes, various vegetables, and fruits [10]. Lignans derived from the roots and rhizomes of certain plants, such as *Podophyllum hexandrum*, have demonstrated potent anticancer activity [11]. In addition, lignans from *Isatis indigotica*, including pinoresinol-*O*-β-d-glucoside, lariciresinol-4-*O*-glucoside, and Clemastanin B, have exhibited anti-influenza virus activity [12–15]. Furthermore, lignans possess medicinal properties, as evident from their antioxidant [16, 17], antibacterial [18], anti-inflammatory [19], and anticoagulant effects [20]. Traditionally, lignans were primarily isolated from plants. However, their limited concentration in plants restricts their broader application [21–24]. For example, sesamin, a multifunctional lignan extracted from sesame oil, which is the richest source of sesamin, constitutes only 0.4–0.6% (w/w) of the oil. However, sesame is planted once annually, which limits its availability in large quantities [22]. Podophyllotoxin, a precursor to semisynthetic antitumor drugs, is extracted from the roots and rhizomes of *Podophyllum hexandrum* and is found in limited areas [25]. Its availability is further threatened by overexploitation and environmental degradation [25]. Thus, developing efficient heterologous synthesis methods for these valuable lignans through metabolic engineering holds substantial economic value.

The biosynthesis of lignans begins with the common phenylpropane pathway, which leads to the formation of its precursor, coniferyl alcohol (Additional file 1: Fig. S1). Subsequently, under the action of dirigent proteins and laccases, oxidative coupling reactions occur to form pinoresinol. Specific enzymes downstream convert pinoresinol into various lignans. The common downstream lignan biosynthetic pathway involves the sequential reduction of pinoresinol to lariciresinol and then to secoisolariciresinol by pinoresinol/lariciresinol reductase (PLR). This is followed by the dehydrogenation of secoisolariciresinol to matairesinol by secoisolariciresinol dehydrogenase (SIRD) [26–29]. However, the downstream lignan synthesis also exhibits species diversity across different plants. To date, lignan biosynthesis pathways have been elucidated for sesamin, forsythin, and podophyllotoxin, among others [30–33].

**Fig. 1:**
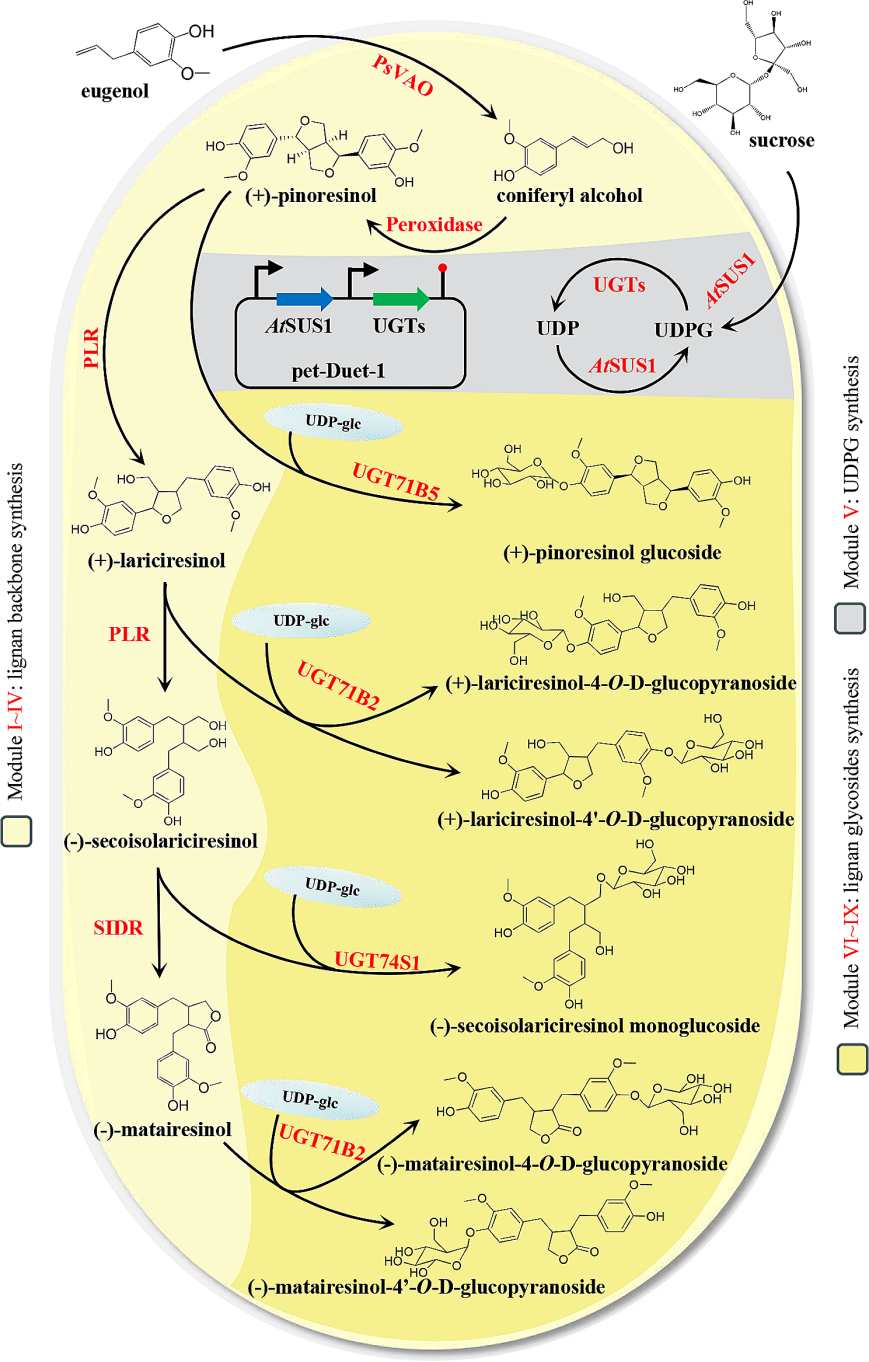
Systematic engineering of *Escherichia coli* metabolism for biosynthesis of lignans and their glucosidated products. Illustration of the modularized platform for producing and exporting lignans and their glucosidated products. Module I ~ IV (lignan skeleton synthesis module) incorporates the synthesis of (+)-pinoresinol using eugenol as substrate, followed by (+)-lariciresinol and (-)-secoisolariciresinol under PLR, and finally (-)-matairesinol under *Pp*SIRD. Module V (UDPG synthesis module ) provides glycoside ligands for producing lignan glycosides. Module VI ~ IX uses the lignan produced with module I ~ IV as glycosylation acceptor and module V as sugar donor to synthesize the corresponding lignan glycosides. *Ps*VAO, Vanillyl alcohol oxidase from *Penicillium simplicissimum*; Prx02, the *E. coli* endogenous peroxidase EcoDyPrx02_536; PLR, pinoresinol-lariciresinol reductases; *Pp*SIRD, Secoisolariciresinol dehydrogenase from *Podophyllum peltatum*; *At*SUS1, sucrose synthase from *Arabidopsis thaliana*; UDPG, uridine diphosphate glucose; *Ii*UGT71B5, UDP-glycosyltransferase 71B5 from *Isatis indigotica*; *Ii*UGT71B2, UDP-glycosyltransferase 71B2 *Isatis indigotica*; *Lu*UGT74S1, UDP-glycosyltransferase 74S1 from *Linum usitatissimum*

Recent advances in synthetic biology have enabled the production of lignans through innovative approaches. Lau et al. used transcriptome mining to identify genes involved in the biosynthesis of podophyllotoxin. By co-expressing 10 genes in tobacco, they successfully reconstructed the pathway to produce (-)-4’-desmethylepipodophyllotoxin [33]. Lv et al. identified two peroxidases and a vanillyl alcohol oxidase in *E. coli* BL21 (DE3), establishing an in vivo enzyme cascade reaction that uses the readily available and cost-effective compound eugenol to produce pinoresinol. This cascade also efficiently removes H_2_O_2_, reducing the toxicity and enzyme inhibition caused by byproducts, thereby facilitating the production of pinoresinol [34]. The yield of lariciresinol was increased to 5.9 g/L through the protein engineering of secoisolariciresinol dehydrogenase [35]. Furthermore, Chen synthesized chiral lignan (-)-lariciresinol glucoside in yeast by using the stereoselective enzymes DIR1/2, PLR, and *Ii*UGT71B2 [36]. Despite these advancements, the synthesis of most lignans, especially the more valuable lignan glycosides, across the entire synthetic network remains challenging. A series of glycosyltransferases (UGT enzymes) responsible for catalyzing lignan glycosylation has been identified in plants. These enzymes exhibit high catalytic activity and specificity, enabling the precise synthesis of lignan glycosides by using different combinations of gene clusters [37–39]. In this study, we explored various enzyme combinations from different sources to achieve the targeted synthesis of compounds along the lignan pathway (Fig. 1). In addition, we investigated the effects of different fermentation methods and genetic structures on product yields, ultimately achieving high yields of the target products.

## Materials and methods

### Chemicals and reagents

The compounds (+)-pinoresinol, (+)-pinoresinol-4-*O*-β-glucopyranoside, pinoresinol diglucoside, (+)-lariciresinol, (-)-secoisolariciresinol, (-)-secoisolariciresinol monoglucoside, (-)-secoisolariciresinol diglucoside, (-)-matairesinol, (-)-matairesinol monoglucoside, and (-)-matairesinoside were purchased from BioBioPha Co., Ltd. (Kunming, China). Eugenol and Clemastanin B were purchased from Yuanye Bio-Technology Co., Ltd. (Shanghai, China). Ethyl acetate was purchased from Sinopharm Chemical Reagent Co., Ltd. (Shanghai, China)

### Construction of strains and plasmids

The heterologous gene sequences used in this study were listed in Table 1 in Additional file 3 Supplementary Data. The codon-optimized genes *Prx02* (http://peroxibase.toulouse.inra.fr/, PeroxiBase Number: 5886) [34, 40], *PsVAO* (NCBI accession number: CAA75722.1), *IiPLR1* (NCBI accession number: AEA42007.1), *TpPLR2* (NCBI accession number: AAF63508.1), *PpSIRD* (NCBI accession number: KR779861.1), *AtSUS1* (NCBI accession number: NM_122090.4) and *LuUGT74S1* (NCBI accession number: JX011632.1) were synthesized by BioSune. Additional gene sequences, including *IiUGT71B5* (NCBI accession number: MW051594) and *IiUGT71B2* (NCBI accession number: MK704396.1), were retrieved from GenBank. The plasmids pET-Duet-1 and pCDF-Duet-1 were used to construct the expression vector. Plasmid propagation was performed in *Escherichia coli* DH5α recipient cells, and recombinant enzyme production was conducted in *E. coli* BL21 (DE3) cells.

The synthesis of (+)-pinoresinol involved inserting two genes, *Prx02* and *PsVAO*, into the multiple cloning site (MCS) 1 of the pCDF-Duet-1 vector (pCDF-Duet-1-Prx02 and pCDF-Duet-1-*Ps*VAO), a process undertaken by BioSune. Then, these two genes were ligated into MCS2 of the pCDF-Duet-1-Prx02 and pCDF-Duet-1-*Ps*VAO vectors and screened on an Luria-Bertani (LB) plate containing 50 µg/µL streptomycin. The resulting plasmids pCDF-Duet-1-Prx02-*Ps*VAO and pCDF-Duet-1-*Ps*VAO-Prx02 were confirmed through Sanger sequencing. *Ii*PLR1 and *Tp*PLR2, which were ligated into MCS1 of pET-Duet-1, were used to synthesize (+)-lariciresinol and (-)-secoisolariciresinol, respectively. *Pp*SIRD was inserted into MCS2 of the pET-Duet-1 for the synthesis of (-)-matairesinol. To synthesize corresponding glycosylation products, *At*SUS1 and UGTs were ligated into MCS1 and MCS2 of pET-Duet-1, respectively. All strains used in this study were listed in Table 2 in Supplementary Data. All primers used in this study were synthesized by BioSune (Shanghai, China) and were listed in Table 3 in Additional file 3 Supplementary Data.

### Biosynthesis of (+)-pinoresinol in *E. Coli*

We synthesized (+)-pinoresinol following a previously described method with some modifications [34]. pCDF-Duet-1-Prx02-*Ps*VAO and pCDF-Duet-1-*Ps*VAO-Prx02 were transformed into *E. coli* BL21 (DE3) cells, and the engineered strains were designated as Str1 and Str2, respectively (Table 2 in Supplementary Data). Positive colonies were selected and cultured at 37 °C and 220 rpm in 15 culture tubes, each containing 5 mL of streptomycin-supplemented LB liquid medium for 16–18 h. Subsequently, 2 mL of the cultured strains were inoculated into 25 mL of terrific broth medium (TB; 12 g/L of tryptone, 24 g/L of yeast extract, and 8 mL/L of 50% glycerol; 1× TB phosphate: 2.31 g KH_2_PO_4_, 12.54 g K_2_HPO_4_) in a 250-mL shaking flask. The cultures were then incubated at 37 °C with shaking at 220 rpm until an OD_600_ of 0.6–0.8 was reached. Protein expression was induced by adding isopropyl-β-d-1-thiogalactopyranoside (IPTG) to a final concentration of 500 µM, followed by cultivation at 25 °C with shaking at 200 rpm for an additional 10–12 h. Bioconversion was initiated at 20 °C with shaking at 100 rpm by adding eugenol to a final concentration of 0.13% (v/v). Subsequently, an additional 0.09% (v/v) eugenol was added every 2 h, reaching a total addition of 0.63% (v/v, or 6.64 g/L) eugenol. Samples were collected every 2 h before feeding and used for analysis.

### Synthesis of lignan downstream products in *E. Coli*

The production of (+)-lariciresinol, (-)-secoisolariciresinol, and (-)-matairesinol involved three stages: the accumulation of pinoresinol, the conversion of (+)-pinoresinol, and the synthesis of (-)-matairesinol. The *E. coli* strain Str1, carrying the plasmid pCDF-Duet-1-Prx02-*Ps*VAO, was used to produce (+)-pinoresinol. For the conversion of (+)-pinoresinol to (+)-lariciresinol and (-)-secoisolariciresinol, we used *E. coli* BL21 (DE3) strains harboring plasmids encoding *Ii*PLR1 and *Tp*PLR2, respectively. The synthesis of (-)-matairesinol was achieved using *E. coli* BL21 (DE3) harboring a plasmid encoding *Pp*SIRD.

Two experimental approaches were explored for the biotransformation of (+)-pinoresinol into (+)-lariciresinol and (-)-secoisolariciresinol. In the “one cell, one-pot” setup, *E. coli* BL21 (DE3) cells co-expressing *Prx02-PsVAO* and *Ii*PLR1 were prepared. Eugenol was added to initiate the reaction. These conversions were conducted in 25 mL of TB medium in 250-mL Erlenmeyer flasks at 20 °C and 100 rpm for 72 h.

Alternatively, the “two-cells, one-pot” approach was used. Initially, *E. coli BL21* (DE3) co-expressing *Prx02-PsVAO* (Module I) was incubated with eugenol in 25-mL culture within 250-mL Erlenmeyer flasks at 20 °C and 100 rpm for 12 h. This was followed by adding 25 mL of *E. coli* BL21 (DE3) with *IiPLR1*/*TpPLR2* (Module II/III), and the reaction was continued for 96 h.

To synthesize (-)-matairesinol, we used two methods. First, we used the mixed “two-cells, one-pot” approach, which involved the accumulation of (+)-pinoresinol for 12 h, followed by the addition of strains co-expressing *Tp*PLR2 and *Pp*SIRD to facilitate biotransformation for 96 h (Module IVa). In addition, the mixed “three-cells, one-pot” approach was used. This approach involved three consecutive steps: the accumulation of (+)-pinoresinol by the *Prx02-PsVAO* strain for 12 h, the accumulation of (-)-secoisolariciresinol by the *Tp*PLR2 strain for 12 h, and the biotransformation of (-)-secoisolariciresinol by the *Pp*SIRD strain for 96 h (Module IVb).

### Whole-cell synthesis of lignan glycosylation products

Lignan glycosides were produced using a one-pot multicellular fermentation process. During the glycosylation process, 14 g/L of sucrose [41] was added to facilitate UDPG regeneration for the glycosylation reaction. (+)-Pinoresinol glucoside (PG, module VI) was produced by mixing modules I and V-A and adding strains co-expressing *At*SUS1 and *Ii*UGT71B5 12 h after the accumulation of (+)-pinoresinol. (+)-Lariciresinol-4’-*O*-d-glucopyranoside and (+)-lariciresinol-4-*O*-d-glucopyranoside (LGs, module VII) were synthesized through one-pot fermentation involving modules I, II, and V-B. This process involved the addition of the strain expressing *Ii*PLR1 to produce (+)-lariciresinol after 12 h of (+)-pinoresinol accumulation, followed by the addition of strains co-expressing *At*SUS1 and *Ii*UGT71B2. (-)-Secoisolariciresinol monoglucoside (SG, module VIII) was formed by first accumulating (+)-pinoresinol with Module I for 12 h. Then, Module III was added to facilitate the accumulation of (-)-secoisolariciresinol. After 12 h, strains co-expressing *At*SUS1 and *Lu*UGT74S1 were added. Finally, (-)-matairesinol-4-*O*-d-glucopyranoside and (-)-matairesinol-4’-*O*-d-glucopyranoside (MGs, module IX) were generated during the fermentation of modules I, III, IVb, and V-B in a four-cell pot, which involved the accumulation of (+)-pinoresinol for 12 h, (-)-secoisolariciresinol for 12 h, and (-)-matairesinol for 12 h, as well as the production of the final glucoside products.

### Ultrahigh Performance Liquid Chromatography–Mass Spectrometry Analysis of products

We extracted (+)-pinoresinol, (+)-lariciresinol, (-)-secoisolariciresinol, and (-)-matairesinol along with their corresponding glycosylation products twice from the cell cultures by using a 1:1 ratio of ethyl acetate to liquid (v/v). The samples were sonicated for 30 min. The mixture was then centrifuged at 7830 rpm for 5 min to separate the two phases. The supernatants from both centrifugation steps were combined, and the solvent was evaporated using a centrifugal concentrator. The concentrated sample was then redissolved in 500 µL of methanol. Subsequently, the samples were centrifuged for 20 min, and the resulting supernatant was transferred to sample vials for measurements.

All products were analyzed using an Agilent 1290 A Infinity II ultra-performance liquid chromatography (UHPLC) system coupled with the Agilent 6530 A accurate-mass quadrupole-time-of-flight mass spectrometer (Q-TOF/MS) (Agilent, USA) equipped with a dual AJS electrospray ionization (ESI) source operated in the negative ion mode. The details of the parameters are provided in Supplementary materials (Additional file 2).

Quantitative analysis was performed using high-performance liquid chromatography (HPLC)–tandem mass spectrometry (MS/MS) with an Agilent 1200 A series liquid chromatograph coupled with an Agilent 6410 A triple-quadrupole mass spectrometer equipped with an ESI source (Agilent, USA). The details of the parameters are provided in Supplementary materials (Additional file 2).

## Results

### Production of (+)-pinoresinol in *E. Coli*

**Fig. 2:**
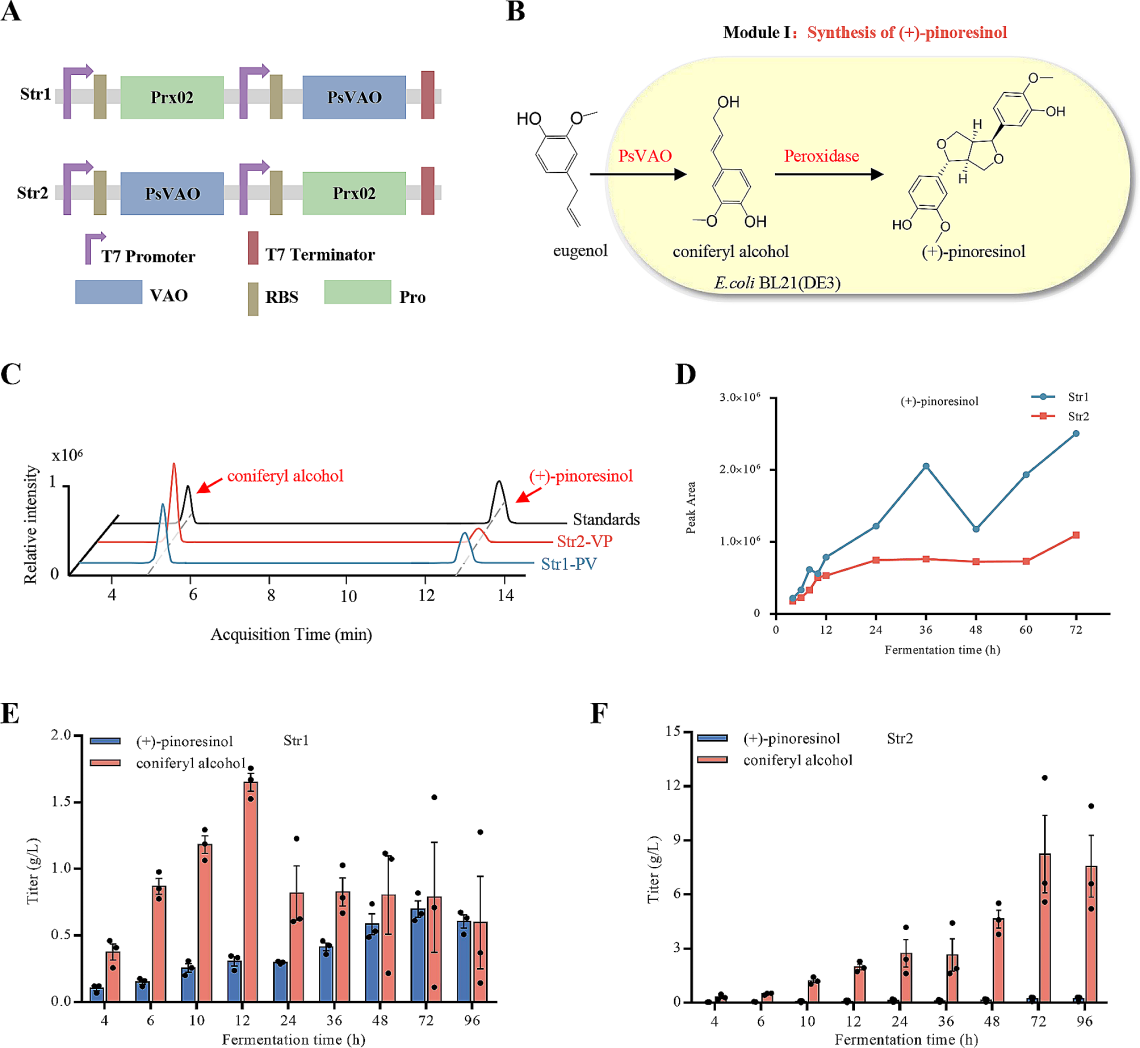
Effects of different gene orders on the production of (+)-pinoresinol in *E. coli*. (**A**) A genetic architecture of different recombinant strains for the (+)-pinoresinol production. (**B**) A schematic of the (+)-pinoresinol production in *E. coli* using unexpensive eugenol as a substrate, known as module I. (**C**) UHPLC spectra of coniferyl alcohol and (+)-pinoresinol produced by Str 1 and Str 2 and their standards. (**D**) The peak area of (+)-pinoresinol production of Str 1 was higher than that of Str 2. (**E**) (+)-Pinoresinol and coniferyl alcohol titer of Str 1. (**F**) (+)-Pinoresinol and coniferyl alcohol titer of Str 2

Product yield is substantially affected by the architectural designs and construction methods used, such as ePathBrick and fusion gene techniques [34, 41]. To investigate the effect of cloning order on product yield, we inserted two genes into different multiple cloning sites to obtain the recombinant plasmids pCDF-Duet-1-Prx02-*Ps*VAO and pCDF-Duet-1-*Ps*VAO-Prx02 (Additional file 3: Table 2 in Supplementary Data). These plasmids were then expressed in *E. coli* BL21(DE3) to construct the corresponding recombinant strains *E. coli* Str1 and Str2 (Fig. 2A, Additional file 3: Table 2 in Supplementary Data). When these strains were cultured with eugenol, they produced (+)-pinoresinol, referred to as “Module I” (Fig. 2B). UHPLC-QTOF-MS analysis revealed two peaks at retention times of 4.8 min (m/z 179.07) and 12.3 min (m/z 357.13), corresponding to coniferyl alcohol and (+)-pinoresinol, respectively, as confirmed by matching fragment ion information with standards (Additional file 1: Fig. S2A, B). Moreover, the peak area was larger for (+)-pinoresinol produced by *E. coli* Str1 than for that produced by *E. coli* Str2 (Fig. 2C and D). Subsequently, the products of the two strains were accurately quantified. The highest titers of (+)-pinoresinol produced by Str1 and Str2 were 698.9 mg/L with a molar yield of 2.5% (Fig. 2E) and 218.7 mg/L with a molar yield of 0.8% (Fig. 2F), respectively. In addition, during the entire fermentation process, the yield of coniferyl alcohol, an intermediate produced by Str1, demonstrated a declining trend after 12 h (Fig. 2E). However, no such declining trend was observed for Str2 (Fig. 2F). Moreover, the accumulation of the intermediate product was higher in Str2 than in Str1 (Additional file 1: Fig. S2C). Thus, we selected Str1 for subsequent experiments.

### In vivo production of (+)-lariciresinol and (-)-secoisolariciresinol in *E. Coli*

**Fig. 3:**
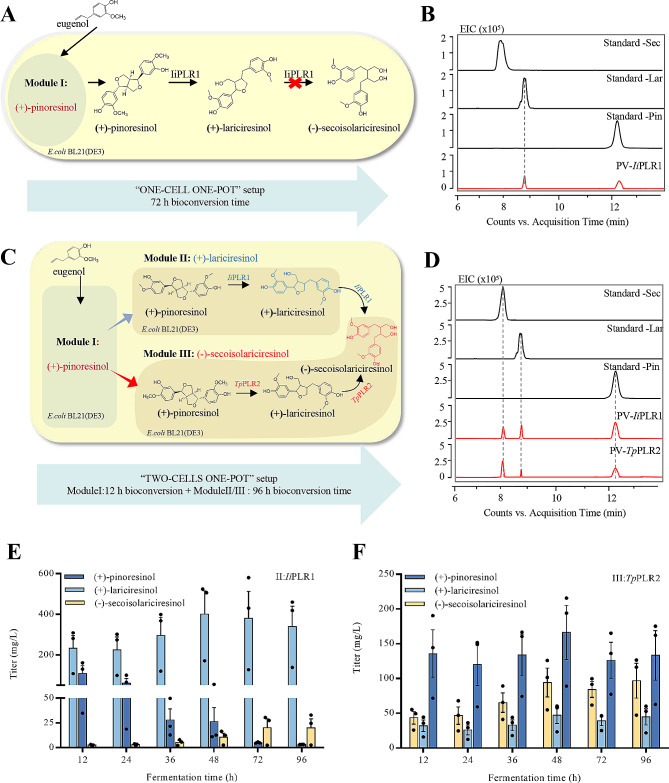
Overview of the two setups used for multistep transformation of (+)-pinoresinol to (+)-lariciresinol and (-)-secoisolariciresinol, respectively. (**A**) “One-cell one pot” setup: *E. coli* BL21(DE3) cells harboring pCDF-Duet-1-Prx02-*Ps*VAO and pET-Duet-*Ii*PLR1 with supplement of eugenol. (**B**) UHPLC-MS/MS chromatograms for production of (+)-lariciresinol as the only product with catalyzing of *Ii*PLR1 by “One-cell one pot” setup. (**C**) Sequential “two cells one-pot” setup: *E. coli* BL21(DE3) cells harboring pCDF-Duet-1-Prx02-*Ps*VAO supplemented with eugenol were incubated for 12 h alone to convert eugenol to (+)-pinoresinol, afterwards *E. coli* BL21(DE3) cells harboring pET-Duet-*Ii*PLR1 or pET-Duet-*Tp*PLR2 were added, and the incubation time was prolonged for 96 h, known as Module II or Module III. (**D**)UHPLC-MS/MS chromatograms for production of (+)-lariciresinol and (-)-secoisolariciresinol with eugenol as substrate by “two cells one-pot” setup; (+)-lariciresinol and (-)-secoisolariciresinol was detected at 12 h. (**E**) The titers of (+)-pinoresinol, (+)-lariciresinol and (-)-secoisolariciresinol produced by module II through “two cells one-pot” setup. (**F**) (+)-Pinoresinol, (+)-lariciresinol and (-)-secoisolariciresinol titer of module III by “two cells one-pot” setup. Pin: (+)-pinoresinol; Lar: (+)-lariciresinol; Sec: (-)-secoisolariciresinol

Two methods were used to synthesize (+)-lariciresinol and (-)-secoisolariciresinol in *E. coli*. First, a “one-cell, one-pot,” strategy was used (Fig. 3A): The pCDF-Duet-1 vectors harboring *Prx02* and *PsVAO* genes and the pET-Duet-1 vectors harboring the *Ii*PLR1 gene were co-transformed into *E. coli* BL21(DE3) to form the Strpviz strain (Additional file 3: Table 2 in Supplementary Data) for shake-flask fermentation. Subsequently, eugenol was added to initiate the reaction. Samples were collected every 12 h for analyses. UHPLC-MS results indicated that a peak, matching the ion information of (+)-lariciresinol standard fragments (m/z 359.15, retention time: 8.6 min), was detectable only after 72 h of the reaction. However, the production of (-)-secoisolariciresinol was not observed (Fig. 3B).

Second, *Ii*PLR1 and *Tp*PLR2 have different substrate preferences that *Ii*PLR1 can convert (+)-pinoresinol to (+)-lariciresinol, while *Tp*PLR2 shows a higher propensity for catalyzing the conversion of (+)-pinoresinol to (-)-secoisolariciresinol [35]. Consequently, these two enzymes were utilized in the synthesis of different final products. Here, we used two mixed “two-cells, one-pot” setups. The pET-Duet-1 plasmid containing *Ii*PLR1 was transformed into *E. coli* BL21(DE3) to form the StrpI1 (Additional file 3: Table 2 in Supplementary Data) strain for Module II, and the pET-Duet-1 plasmid containing *Tp*PLR2 was transformed into *E. coli* BL21(DE3) to form the StrpT2 strain (Additional file 3: Table 2 in Supplementary Data) for Module III. Eugenol was added to Module I and allowed to react for 12 h. Subsequently, either Module II or Module III was added, and the reaction was extended for 96 h (Fig. 3C). The peak for (+)-lariciresinol (m/z 359.15) was detected at RT 8.6 min (Fig. 3D, Additional file 1: Fig. S3A), and another peak, at retention time of 8 min, was identied as (-)-secoisolariciresinol (m/z 361.16) (Fig. 3D, Additional file 1: Fig. S3B). The results indicated that the peak area for (+)-lariciresinol was larger when Module II was mixed in than when Module III was mixed in. By contrast, the peak area for (-)-secoisolariciresinol was smaller than that observed with Module III (Fig. 3D), indicating that *Ii*PLR1 was more likely to produce (+)-lariciresinol and *Tp*PLR2 was more likely to produce (-)-secoisolariciresinol. Subsequently, quantitative analysis of the fermentation products revealed that the concentration of (+)-lariciresinol reached its peak at 434.08 mg/L after 48 h with the inclusion of Module II (Fig. 3E), whereas the highest concentration of (-)-secoisolariciresinol was 20.17 mg/L. Following the mixed fermentation of Module III and Module I, the highest concentration of (-)-secoisolariciresinol reached 96.81 mg/L, whereas the highest concentration of (+)-lariciresinol was 47.21 mg/L (Fig. 3F). These findings align with the earlier UHPLC–MS observations.

### Whole-cell synthesis of (-)-matairesinol in *E. Coli*

To synthesize (-)-matairesinol, we used the “two-cells, one-pot” setup. As illustrated in Fig. S4A in Additional file 1, we used Module I for the accumulation of (+)-pinoresinol. Subsequently, the pET-Duet-1 plasmid containing *Tp*PLR2 and *Pp*SIRD genes was co-expressed and transformed into BL21(DE3) to form the StrTpSD strain (Module IVa, Additional file 3: Table 2 in Supplementary Data). This strain was combined with Module I in a shake-flask fermentation process to produce (-)-matairesinol. UHPLC–MS analysis of the fermentation products revealed the presence of (+)-lariciresinol, (-)-secoisolariciresinol, and (+)-pinoresinol at retention times of 8.6, 8, and 12.3 min, respectively. However, the target product (-)-matairesinol was not detected (Additional file 1: Fig. S4B).

**Fig. 4:**
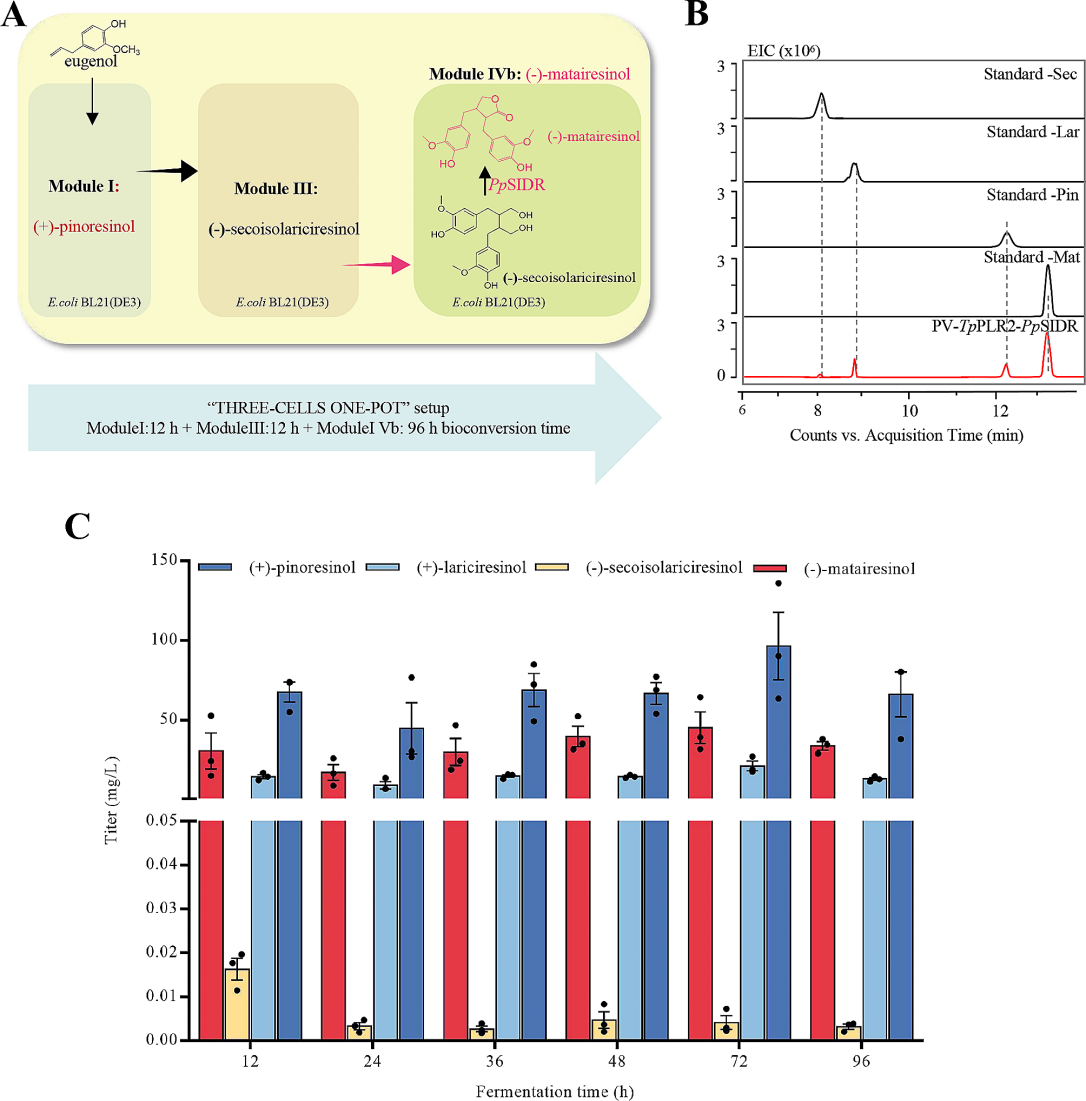
Multisteps synthesis of (-)-matairesinol in *E. coli*. (**A**) Sequential “three cells one-pot” setup: module I was used for the production of (+)-pinoresinol, afterwards 12 h, module III was added, and the incubation time was prolonged for 12 h, and last, *E. coli* strain harboring pET-Duet-*Pp*SIRD were added (module IVb) for fermentation of 96 h. (**B**) UHPLC chromatograms for production of (-)-matairesinol by “three cells one-pot” setup; desired product (-)-matairesinol was detected after adding module IVb 12 h, and meanwhile, the intermediate product (+)-pinoresinol, (+)-lariciresinol and (-)-secoisolariciresinol were also detected. (**C**) (+)-Pinoresinol, (+)-lariciresinol, (-)-secoisolariciresinol and (-)-matairesinol titer of module IVb by “three cells one-pot” setup. Pin: (+)-pinoresinol; Lar: (+)-lariciresinol; Sec: (-)-secoisolariciresinol; Mat: (-)-matairesinol

We shifted to a “three-cell, one-pot” strategy for the in vivo synthesis of (-)-matairesinol in *E. coli* (Fig. 4A). Following the previous approach, we used Module I to accumulate (+)-pinoresinol. We employed *E. coli* BL21(DE3) containing the *Tp*PLR2 gene as Module III to accumulate (-)-secoisolariciresinol, which is a precursor of (-)-matairesinol. Subsequently, *E. coli* BL21(DE3) expressing the gene *Pp*SIRD was used as Module IVb for the transformation of (-)-secoisolariciresinol. As depicted in Fig. 4B, we observed a peak at a retention time of 13.3 min (m/z 357.13), corresponding to the ion profile of (-)-matairesinol standard fragments (Additional file 1: Fig. S4C). In addition, we detected the intermediate products, namely (-)-secoisolariciresinol, (+)-lariciresinol, and (+)-pinoresinol. Quantitative analysis revealed that the highest concentration of (-)-matairesinol achieved through co-culture was 45.14 mg/L (Fig. 4C). The trend showing the variations in the output of intermediate products was depicted in Fig. S4D in Additional file 1. The remaining concentration of the precursor (-)-secoisolariciresinol was the lowest, followed by those of the intermediate products (+)-lariciresinol and (+)-pinoresinol.

### Construction of the UDPG sugar donor module in *E. Coli*

**Fig. 5:**
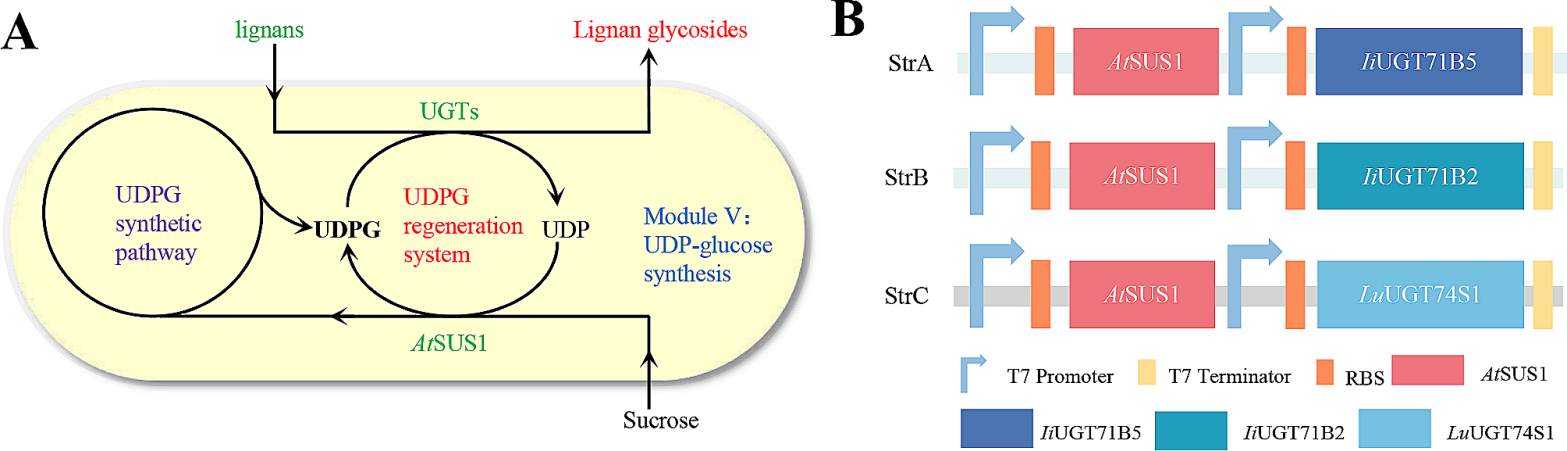
A combination of UDPG and lignan sugar acceptors synthesis module to produce the corresponding lignan glycosides in *E. coli*. (**A**) Sucrose was used as the sugar donor for the bioproduction of Lignan glycosides through the engineered UDPG regeneration system. (**B**) A genetic architecture of different recombinant strains for the different lignan glycosides productions.StrA was used for the (+)-pinoresinol glucoside (PG). StrB was used for the production of (+)-lariciresinol-4’-*O*-d-glucopyranoside, (+)-lariciresinol-4-*O*-d-glucopyranoside (LGs), and (-)-matairesinol-4-*O*-d-glucopyranoside and (-)-matairesinol-4’-*O*-d-glucopyranoside (MGs). StrC was used for producing (-)-secoisolariciresinol monoglucoside (SG)

UGTs have been widely used for the glycosylation of multiple natural products. However, the high cost of sugar donor UDPG required for this process poses a challenge for the cost-effective, whole-cell synthesis of lignan glycosides in *E. coli* [42]. To address this issue, we constructed a self-sustaining UDPG system in engineered *E. coli* by coupling UGT from different species with sucrose synthase (SUS1) (Fig. 5A) [41, 43]. The construction of UGT coupled with SUS1 is depicted in Fig. 5B. The pET-Duet-1 plasmid containing *At*SUS1 and *Ii*UGT71B5 genes was transformed into *E. coli* BL21(DE3) to form the strain StrAIB5 (Additional file 3: Table 2 in Supplementary Data). This strain was used as the UDPG Module V-A in the synthesis of PG products. We substituted *Ii*UGT71B5 with *Ii*UGT71B2 and *Lu*UGT74S1 to create strains StrAIB2 and StrALS1 (Additional file 3: Table 2 in Supplementary Data), respectively. The strain StrAIB2 was used as UDPG Module V-B for the synthesis of lignan glycosides (LGs) and monoglucosides (MGs), whereas StrALS1 was used as UDPG Module V-C for the synthesis of (-)-secoisolariciresinol monoglucoside (SG) (Additional file 1: Fig. S5).

### Synthesis of six lignan glycosylation products in *E. Coli*

Multi-strain mixed fermentation was performed to synthesize six lignan glycosylation products by combining the various aforementioned modules. The process was conducted as follows: eugenol was converted using Module I for 12 h. Subsequently, Module V-A and 14 g/L sucrose were added, and the conversion reaction was extended to 96 h (Additional file 1: Fig. S5A). After eugenol underwent a 12-h reaction with Module I, Module II was added, and the reaction was conducted for an additional 12 h. Then, the UDPG Module V-B and 14 g/L sucrose were added to produce LGs for 96 h (Additional file 1: Fig. S5B). Following the strategy used for (-)-secoisolariciresinol synthesis, SG was produced by adding UDPG Module V-C and 14 g/L sucrose for 96 h after combining Module III with Module I for 12 h (Additional file 1: Fig. S5C). After eugenol underwent a 12-h reaction with Module I, Module III was added for the production of (-)-secoisolariciresinol. After another 12-h reaction, Module IV was added for the production of (-)-matairesinol. Then, after 6 h, UDPG Module V-B and 14 g/L sucrose were added to synthesize MGs for 96 h (Additional file 1: Fig. S5D).

**Fig. 6:**
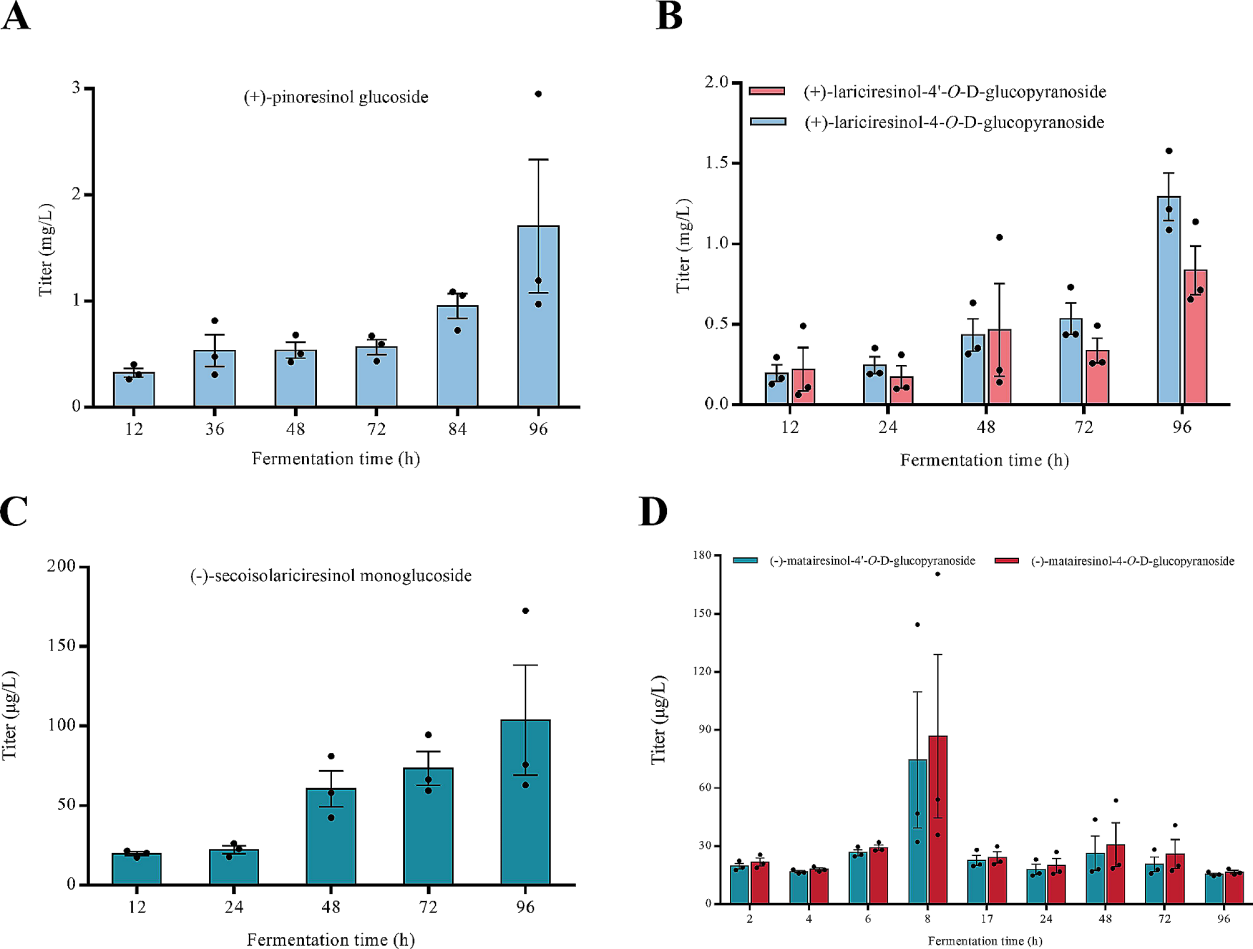
The titer of (**A**) (+)-pinoresinol glucoside (PG), (**B**) (+)-lariciresinol-4’-*O*-d-glucopyranoside and (+)-lariciresinol-4-*O*-d-glucopyranoside (LGs), (**C**) (-)-secoisolariciresinol monoglucoside (SG) and (**D**) (-)-matairesinol-4-*O*-d-glucopyranoside and (-)-matairesinol-4’-*O*-d-glucopyranoside (MGs)

UHPLC–MS analysis of the co-culture fermentation products revealed a peak (m/z = 519.18) at a retention time of 6 min, consistent with the ion profile of PG standard fragments (Additional file 1: Fig. S5A, S6A). Quantitative analysis revealed that the maximum titer of PG obtained through mixed fermentation was 1.71 mg/L (Fig. 6A). At a retention time of 4.5 min, a peak (m/z = 521.20) consistent with the ion profile of (+)-lariciresinol-4’-*O*-d-glucopyranoside standard fragments was detected (Additional file 1: Fig. S5B, S6B). In addition, a peak consistent with the ion profile of (+)-lariciresinol-4’-*O*-d-glucopyranoside fragments was detected at a retention time of 5 min. This peak was identified as (+)-lariciresinol-4-*O*-d-glucopyranoside (Additional file 1: Fig. S6C). The highest titers of LGs obtained through mixed fermentation were 1.3 mg/L for (+)-lariciresinol-4-*O*-d-glucopyranoside and 836 µg/L for (+)-lariciresinol-4’-*O*-d-glucopyranoside (Fig. 6B). At a retention time of 5.5 min, we detected a peak (m/z = 523.21) consistent with the ion profile of the SG standard fragment (Additional file 1: Fig. S5C and Fig. S6D). The highest SG titer obtained through mixed fermentation was 103.77 µg/L (Fig. 6C). Furthermore, we detected peaks (m/z = 519.18) consistent with the ion profiles of (-)-matairesinol-4-*O*-d-glucopyranoside and (-)-matairesinol-4’-*O*-d-glucopyranoside standard fragments at retention times of 7.3 and 7.5 min, respectively (Additional file 1: Fig. S5D, S6E, and S6 F). The highest titers of (-)-matairesinol-4-*O*-d-glucopyranoside and (-)-matairesinol-4’-*O*-d-glucopyranoside obtained through mixed fermentation were 86.79 and 74.5 µg/L, respectively (Fig. 6D).

## Discussion

Lignans possess a broad range of pharmacological activities. The synthesis pathway of lignan in *I. indigotica* Fort has been elucidated [37], and studies have synthesized some products of the lignan pathway in vivo [34, 36, 44]. However, no study has yet completely replicated the lignan biosynthesis pathway in *E. coli*. Here, we used Lv’s method to synthesize lignans using inexpensive eugenol as a precursor in *E. coli* [34]. Our results revealed that in our engineered *E. coli* system, the Str1 strain was the most effective for the in vivo synthesis of (+)-pinoresinol (Additional file 1: Fig. S7). However, the conversion rate of eugenol to pinoresinol was low, which was only 2.5%. There were two possible reasons for this. First, a large number of products remain in the intermediate product coniferyl alcohol, indicating that the catalytic efficiency of *Ps*VAO is higher than that of Peroxidase. We have tried different construction methods and genes fusion expression, but the conversion rate cannot be effectively improved. This suggests that we may need to improve the conversion efficiency by replacing Peroxidase from other sources or modifying Peroxidase, optimizing the fermentation conditions and feeding precursor. Furthermore, on analyzing the fermentation products, we observed that the production of (+)-pinoresinol in this system was accompanied by the appearance of its isomers (Additional file 1: Fig. S8) which can also affects the efficient utilization of the eugenol. However, we did not observe the production of the corresponding byproducts of dehydrodiconiferyl alcohol in the downstream lignan synthesis pathway. These findings indicated that PLR catalyzes only the products with an 8–8’ linkage, thus avoiding the complication of downstream products. This also suggested that module I has further optimization potential, which can be achieved by suppressing such by-products to enhance the conversion of the target product.

Building on the consistent production of (+)-pinoresinol using our engineered *E. coli* system, we synthesized the downstream lignan pathway products in *E. coli*. Different fermentation methods, such as “one-cell, one-pot” and “two-cells, one-pot” affect the generation of products [44]. Ricklefs et al. found that the “two-cells, one-pot” approach was more conducive to the generation of target products compared to the “one-cell, one-pot” approach [45], and authors speculated that this phenomenon might be due to the accumulation of intermediates/by-products or the toxicity of eugenol under these reaction conditions, which could be toxic to *E. coli* cells, or could negatively affect the activity of the enzymes involved in the second step, thereby preventing the completion of the second step reaction in vivo. Similar to the results of Ricklefs et al., we speculate that the “one-cell, one-pot” approach which all three enzymes react simultaneously within *E. coli*, the cascade reaction of the first step *Ps*VAO and Prx02 was not affected by the by-products or substrate toxicity generated during fermentation. However, in the second step reaction, the activity of *Ii*PLR1 enzyme responsible for converting (+)-pinoresinol to (+)-lariciresinol and (-)-secoisolariciresinol was affected by these factors, resulting in its failure to convert (+)-pinoresinol [45]. Detecting (-)-secoisolariciresinol may require even longer fermentation times, which was not conducive to the production of subsequent products. While, the “two-cells, one-pot” approach was more effective for synthesizing multiple pathway products. Therefore, for the in vivo synthesis of subsequent products such as (-)-matairesinol and glycosylation derivatives, we continued to employ co-culture fermentation.

With the gradual increase in the number of co-culture strains, we observed a gradual decline in the production from (+)-pinoresinol to (-)-matairesinol. This decrease could be attributed to the decreased vitality of the strains with an increase in the number of strains involved in mixed fermentation and the prolonged fermentation period, preventing the products from being produced under optimal strain conditions. To address this, we can improve the production of lignan products by making strategic modifications to *E. coli* strains through metabolic engineering [46]. In addition, several genetic strategies can be used to redirect metabolic flux toward the production of desired metabolites. These strategies include increasing the availability of precursors, overexpressing or enhancing the efficiency of bottleneck enzymes, altering the regulation of gene expression, and reducing the flux of unwanted byproducts or competing pathways [46]. Watanabe et al. successfully achieved the complete biosynthesis of the antitumor nonribosomal peptide acanthomycin in *E. coli* by using a three-plasmid system. This system incorporated the acanthomycin biosynthesis gene from *Streptomyces rasamensis*, *sfp*; fabC that encodes a fatty acyl carrier protein; and a gene that confers acanthomycin resistance [47]. Following this approach, we introduced three plasmids into *E. coli*: pCDF-Duet-1-Prx02-*Ps*VAO, pET-Duet-1-*Ii*PLR1/*Tp*PLR2, and pET-Duet-1-*Pp*SIRD. These plasmids were designed to reduce the proliferation of co-culture strains, thereby reducing the decline in catalytic activity due to reduced strain activity over prolonged fermentation times. In another instance of rational engineering, homologous guide point mutations were used to modify the active site of L-equadiene synthetase, enhancing its productivity [48]. We could modify the PLR enzyme, which catalyzes the generation of (+)-lariciresinol or (-)-secoisolariciresinol [35], to enhance its catalytic ability and thus increase product yield.

There is currently limited research that can achieve the directed synthesis of final products on each branch of a metabolic network through metabolic engineering. Although modularity is a common research strategy in synthetic biology, successful examples are relatively few. Recently, Yao et al. established a multi-enzyme one-pot cascade reaction system, which, by introducing different enzymes and building blocks, achieved the combinatorial synthesis of 26 phenylethanol glycosides [49]. This demonstrates that in order to efficiently achieve modular synthesis, it is still necessary to rely on deep pathway analysis and the accumulation of diverse catalytic elements. During the synthesis of LGs in vivo, we used the co-culture fermentation method. To utilize the well functionalized UGTs involved in lignan metabolism [37–39], we can achieve biosynthesis of almost all lignan glycosides. During glycosylation, the inclusion of UDPG is essential for the reaction, and the formation of glycoside products can be enhanced through a cascade reaction involving *At*SUS1 and UGTs (Additional file 1: Fig. S9). However, the yield of glycosylated products obtained through mixed fermentation was lower than that of lignan products. To address this issue, in addition to enhancing lignan products as described earlier, we modified UGT enzymes based on previous studies on early structural biology [37, 38]. Use of the modified enzymes enhanced the in vivo production of lignan glycoside products, thereby improving the yield of LGs. Furthermore, we plan to synthesize lignan bisaccharides in vivo through enzyme modification, marking a step forward toward achieving our objectives.

## Conclusions

We used various construction and fermentation methods for the synthesis of lignan synthesis pathway products in *I. indigotica* Fort and compared their efficiency. The “multicellular one-pot” method proved to be effective for producing multiple products, facilitating the whole-cell synthesis of 10 products within the lignan pathway in *I. indigotica* Fort using *E. coli*. By strategically segmenting and integrating different modules, we can achieve the specific production among the complex lignan biosynthesis net work. The framework established in this research lays a groundwork for the synthesis of products along other lignan pathways and for futher higher product yields. The ability to produce economically and medicinally valuable lignans through the bioconversion of affordable eugenol in *E. coli* is highly crucial.

### Electronic supplementary material

Below is the link to the electronic supplementary material.

Supplementary Material 1

Supplementary Material 2

Supplementary Material 3

## Data Availability

All data generated or analyzed during this study are included in this published article and its supplementary information files.

## Abbreviations

*E. coli*: *Escherichia coli*
PLR: Pinoresinol/lariciresinol reductase
SIRD: Secoisolariciresinol dehydrogenase
PG: (+)-Pinoresinol glucoside
LGs: (+)-Lariciresinol-4’-*O*-d-glucopyranoside and (+)-lariciresinol-4-*O*-d-glucopyranoside
SG: (-)-Secoisolariciresinol monoglucoside
MGs: (-)-Matairesinol-4-*O*-d-glucopyranoside and (-)-matairesinol-4’-*O*-d-glucopyranoside
UHPLC–MS: Ultra-High-Performance Liquid Chromatography–Mass Spectrometry
UHPLC: Ultra-performance liquid chromatography
TOF/MS: Quadrupole-time-of-flight mass spectrometer
ESI: Electrospray ionization source
UDPG: uridine diphosphate glucose
UGT: UDP-glucose transferase
SUS1: Sucrose synthase
NMR: Nuclear magnetic resonance
