# Supplementary material for: Construction of lignan glycosides biosynthetic network in Escherichia coli using mutltienzyme modules
Source: Microb Cell Fact. 2024 Jul 5;23:193. doi: 10.1186/s12934-024-02467-1 (PMC11225284; doi:10.1186/s12934-024-02467-1)
Supplement: Supplementary file 2 — Supplementary Material 2 [file 12934_2024_2467_MOESM2_ESM.doc]

Supplementary materials and methods

2.7 Ultrahigh Performance Liquid Chromatography–Mass Spectrometry Analysis of Products

The UHPLC-Q-TOF/MS parameters were as follows: nitrogen drying gas temperature, 350°C; flow, 11 L/min; nebulizer pressure, 45 psi; the sheath gas temperature and flow rate were the same as those of the drying gas; capillary voltage, 4 kV; fragmentor voltage, 120 V; skimmer voltage, 60 V; octopole 1-RF peak voltage, 750 V; and mass range, 100–3200 m/z. Chromatographic separation was performed using an InfinityLab Poroshell 120 EC-C18 column (2.7 µm, 3.0 × 150 mm; Agilent) at 50°C, and the mobile phase consisted of 0.01% formic acid and acetonitrile (ACN). Then, an elution method was used as follows: 5% ACN at 0 min, 20% ACN at 2 min, 25% ACN at 10 min, 95% ACN at 20 min, and a final 4.5 min of equilibration postrun. The injection volume was 2.0 μL, and the flow rate was 0.3 mL min^-1^. All data acquisition and analyses were performed using Agilent MassHunter Workstation Software Qualitative Analysis, Version B.03.01 (Agilent Technologies, USA).

The parameters of 1200-6410 LC/MS quantitative analysis were as follows: Chromatographic separation was performed on a ZORBAX SB-C18 column (3.5 µm, 2.1 × 100 mm; Agilent) at 30°C. The mobile phase consisted of 5 mM ammonium acetate in water and ACN. Then, a gradient elution method was used as follows: 14% ACN at 0 min, 50% ACN at 6 min, and 85% ACN at 6.5–12 min. The flow rate was 0.3 mL min^-1^.
