# Supplementary material for: Construction of lignan glycosides biosynthetic network in Escherichia coli using mutltienzyme modules
Source: Microb Cell Fact. 2024 Jul 5;23:193. doi: 10.1186/s12934-024-02467-1 (PMC11225284; doi:10.1186/s12934-024-02467-1)
Supplement: Supplementary file 1 — Supplementary Material 1 [file 12934_2024_2467_MOESM1_ESM.doc]

**Fig. S1** Lignan biosynthetic pathways. PAL, phenylalanine ammonia-lyase; C4H: cinnamate 4-hydroxylase; 4CL, 4-coumarate:coenzyme A ligase, C3H: coumarate-3-hydroxylase; CCoAOMT, caffeoyl coenzyme A-O-methyltransferase; CCR, cinnamoyl-CoA reductase; CAD, cinnamyl alcohol dehydrogenase; DIR, dirigent protein; PLR, pinoresinol–lariciresinol reductases; SIRD, secoisolariciresinol dehydrogenase; UGT, uridine diphosphatedependent glycosyltransferase.

**Fig. S2** Identification of metabolites produced by the module I. **(A)** MS/MS analyses of coniferyl alcohol. **(B)** MS/MS analyses of (+)-pinoresinol. ^*^ *P*<0.05 compared with the Str1; ^**^ *P*<0.01 compared with the Str1. **(C)** Titer of coniferyl alcohol [produced in different strains.

**Fig. S3** Identification of metabolites produced by the module II and III. **(A)** MS/MS analyses of (+)-lariciresinol. **(B)** MS/MS analyses of (-)-secoisolariciresinol.

**Fig. S4** Synthesis of (-)-matairesinol in *E. coli* by multisteps*.* **(A)** Sequential “two cells one-pot” setup: module I was used for the producing (+)-pinoresinol, afterwards 12 h, module IVa was added, and the incubation time was prolonged to 96 h. **(B)** UHPLC chromatograms for production of (-)-matairesinol by “two cells one-pot” setup; desired product (-)-matairesinol was not detected. **(C)** MS/MS analyses of (-)-matairesinol. **(D)** The titer variation trend of each product during the synthesis of (-)-matairesinol. Pin: (+)-pinoresinol; Lar: (+)-lariciresinol; Sec: (-)-secoisolariciresinol; Mat: (-)-matairesinol.

**Fig. S5** Synthesis of lignan glycosides in *E. coli.* **(A)** “Two cells one pot” setup including module I and UDPG module V-A was used to produce (+)-pinoresinol glycoside; UHPLC chromatograms for production of (+)-pinoresinol glucoside was also shown on the right. **(B)** “Three cells one pot” setup including module I, module II and UDPG module V-B was used to produce (+)-lariciresinol-4'-*O*-d-glucopyranoside and (+)-lariciresinol-4-*O*-d-glucopyranoside (LGs); UHPLC chromatograms for production of LGs was also showed on the right. **(C)** “Three cells one pot” setup including module I, module III and UDPG module V-C was used for the production of (-)-secoisolariciresinol monoglucoside (SG); UHPLC chromatograms for production of SG can be found on the right. **(D)** “Four cells one pot” setup including module I, module III, module IVb and UDPG module V-B was used for the production of (-)-matairesinol-4-*O*-d-glucopyranoside and (-)-matairesinol-4'-*O*-d-glucopyranoside (MGs); UHPLC chromatograms for production of MGs was also showed in right.

**Fig. S6** Identification of the lignan glycosidation products. MS/MS analyses of each product were represented. **(A)** (+)-pinoresinol glucoside, **(B)** (+)-lariciresinol-4'-*O*-d-glucopyranoside, **(C)** (+)-lariciresinol-4-*O*-d-glucopyranoside, **(D)** (-)-secoisolariciresinol monoglucoside, **(E)** (-)-matairesinol-4'-*O*-d-glucopyranoside, and **(F)** (-)-matairesinol-4-*O*-d-glucopyranoside.

**Fig. S7** Titer of pinoresinol and coniferyl alcohol by recombinant strains harboring different architecture. **(A)** Genetic architecture of different recombinant strains. **(B)** Titler of (+)-pinoresinol and coniferyl alcohol.

**Fig. S8** Identification of (+)-pinoresinol isomers. **(A)** Structural formula of dehydrodiconiferyl alcohol. **(B)** ^1^H-NMR and **(C)** ^13^C-NMR spectrum of dehydrodiconiferyl alcohol. **(D)** MS/MS analyses of dehydrodiconiferyl alcohol.

**Fig. S9** UPLC detection of standards and products of the lignan glycosidation without *At*SUS1 in UDPG module.
